# Application of Real-Time Visual Feedback System in Balance Training of the Center of Pressure with Smart Wearable Devices

**DOI:** 10.3390/ijerph18189637

**Published:** 2021-09-13

**Authors:** I-Lin Wang, Li-I Wang, Yang Liu, Yu Su, Shun Yao, Chun-Sheng Ho

**Affiliations:** 1 College of Physical Education, Hubei Normal University, Huangshi 435002, China; ilin@gms.ndhu.edu.tw; 2 Department of Physical Education and Kinesiology, National Dong Hwa University, Hualien 97046, Taiwan; tennis01@gms.ndhu.edu.tw; 3 Graduate Institute, Jilin Sport University, No. 2476, Freedom Road, Nanguan District, Changchun 130022, China; ly972226173@gmail.com (Y.L.); y.su825@gmail.com (Y.S.); yaoshun0330@gmail.com (S.Y.); 4 Division of Physical Medicine and Rehabilitation, Lo-Hsu Medical Foundation, Inc., Lotung Poh-Ai Hospital, Yilan City 26546, Taiwan; 5 Department of Physical Therapy, College of Medical and Health Science, Asia University, Taichung 41354, Taiwan

**Keywords:** balance training, real-time visual feedback, smart wearable devices, center of pressure

## Abstract

Balance control with an upright posture is affected by many factors. This study was undertaken to investigate the effects of real-time visual feedback training, provided by smart wearable devices for COP changes for healthy females, on static stance. Thirty healthy female college students were randomly divided into three groups (visual feedback balance training group, non-visual feedback balance training group, and control group). Enhanced visual feedback on the screen appeared in different directions, in the form of fluctuations; the visual feedback balance training group received real-time visual feedback from the Podoon APP for training, while the non-visual feedback balance training group only performed an open-eye balance, without receiving real-time visual feedback. The control group did not do any balance training. The balance training lasted 4 weeks, three times a week for 30 min each time with 1–2 day intervals. After four weeks of balance training, the results showed that the stability of human posture control improved for the one leg static stance for the visual feedback balance training group with smart wearable devices. The parameters of COP max displacement, COP velocity, COP radius, and COP area in the visual feedback balance training group were significantly decreased in the one leg stance (*p* < 0.05). The results showed that the COP real-time visual feedback training provided by smart wearable devices can better reduce postural sway and improve body balance ability than general training, when standing quietly.

## 1. Introduction

Balance ability refers to the human body’s ability to adjust automatically to maintain postural stability when it moves or is subjected to external forces [1]. Balance control is usually affected by joint range of motion and muscle strength, which can be used to monitor the sensory information of the mechanism [2]. Therefore, good balance must be regulated by the sensory system and neuromuscular system. During upright posture control, people are clearly aware of their positional changes in space when they are given visual feedback (VF) based on the displacement of the center of pressure (COP) or body center of mass (COM) [3]. A visual system can provide the human body with information about the surrounding environment, location, direction, and speed during movement. When the visual information is removed or altered, the action system must rely on proprioceptive feedback and sensory information from the vestibular system in order to maintain balance [4]. Therefore, VF can help increase the body’s stability and balance ability, while controlling the posture of the human body. 

In recent years, sensorimotor integration technology has been used to provide VF to improve the balance ability of people with disabilities and at high-risk of falls. Previous studies have indicated that internal feedback on one’s own postural sway can be obtained through VF, so that the body can control its posture changes more autonomously [5]. In a study detailing the effect of VF from COP on the balance posture control of adolescents and the elderly, it was found that the use of VF for COP in the standing task is a common method for evaluating and training posture control [6]. Therefore, VF can improve upright posture control and change postural sway in the anterior–posterior and medial–lateral directions to maintain balance. In addition, further findings on ankle movement clarify the effect of different types of VF on body sway and ankle joint mechanisms that contribute to postural sway control [7]. A comparison between traditional body training and computer vision feedback training indicated that the computer vision feedback group had better effects on the balance posture control of the human body [8]. Therefore, providing VF in balance training can effectively improve the balancing ability of participants. 

VF training to control body posture helps improve the body’s ability to maintain balance and achieve stable standing. It can stabilize the body posture and significantly improve static and dynamic balance ability [9]. Previous studies have found that COP displacement and the mean velocity of patients with spinal cord injury decreased after VF standing balance training, indicating that the ability of static and dynamic stability improved significantly after training [9]. After applying wearable devices to balance training for the elderly, the COP area and COP parameters displayed a significant decrease, indicating that balance training is effective for improving postural control and functional performance in older adults [10]. These studies used visual feedback via a display of COP displacements as balance training to maintain stability. Therefore, appropriate external real-time VF information (the position of the real-time COP) should be provided during balance training to improve the control ability of postural balance and increase the benefit of training. In summary, effectively using real-time VF information of COP provided by smart wearable devices for balance control and training can benefit technology-assisted balance training at home, thereby aiding sports training and physical rehabilitation. 

Our previous research explored the immediate effect of visual feedback provided by intelligent devices on the posture control of different genders [11]. It was found that visual feedback balance training with smart wearable devices can improve balance and there are differences in the visual feedback with smart wearable devices between males and females. After visual feedback balance training, females have better balance control ability than males. Therefore, this study aimed to explore the balance improvement of healthy females after visual feedback balance training in the one leg stance (OLS) or tandem stance (TS) postures.

## 2. Materials and Methods

### 2.1. Participants

Thirty healthy female college students were recruited and randomly assigned to the visual feedback balance training group (VFT), non-visual feedback balance training group (NVFT), or no balance training control group (CG), with 10 persons in each group. The basic characteristics of the participants, concerning age (21.30 ± 0.82, 21.44 ± 1.01, and 21.60 ± 1.71 years, respectively), height (168.10 ± 5.63, 164.67 ± 4.68, and 163.90 ± 3.75 cm, respectively), and weight (58.60 ± 6.60, 57.89 ± 7.17, and 56.00 ± 6.82 kg, respectively) in the VFT, NVFT, and CG groups were recorded. Meanwhile, there were no significant differences among the three groups. Exclusion criteria: any past history of injury or treatment of the lower limbs, any neurological or vascular deficit affecting balance, pain and swelling near the ankle and foot, or visual or vestibular impairment [12]. The participants were informed of the content, process, and precautions for the study group. The test instructions were read out to them and they understood and were willing to cooperate fully with the experimenter and signed the consent form. The study was approved by the Research Ethics Committee of Hualien Tzu Chi Hospital, Buddhist Tzu Chi Medical Foundation (IRB109-053-B) and was conducted in accordance with the Declaration of Helsinki.

### 2.2. Equipment

#### 2.2.1. Evaluation Equipment

**Figure 1:**
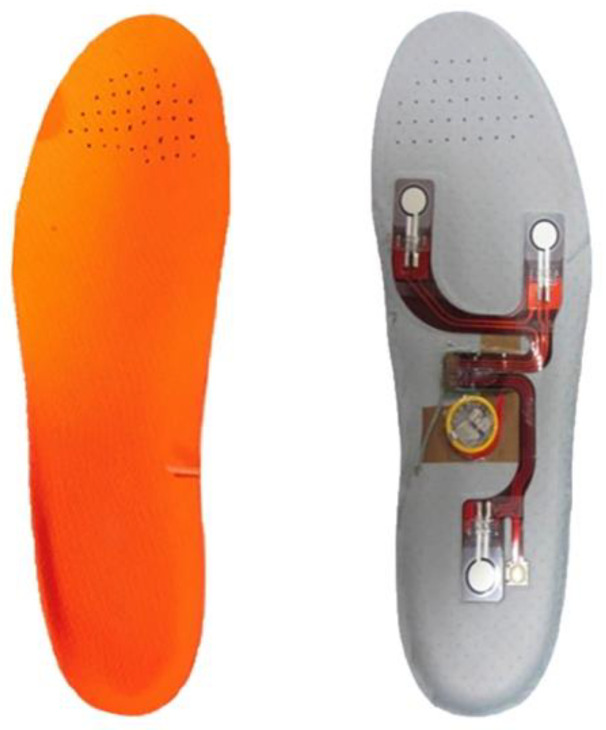
Smart insoles.

A force plate (BTS P6000, BTS Bioengineering, Italy) was used to calculate kinematic data: COP anteroposterior max displacement, COP mediolateral max dis-placement, COP anteroposterior velocity, COP mediolateral velocity, COP radius, and COP area. The force plate signals were collected at a sampling frequency of 300 Hz. In order to avoid the impact of different wear and tear during the test, all participants wore the same experimental tights and uniform sports shoes. The sports shoe incorporated a smart insole (Figure 1). The smart insole was linked to the Podoon APP in an iPad Pro (Apple, Cupertino, CA) to act as a smart wearable device to record COP changes. Podoon APP displays the dynamic points of COP, and participants tried to keep the dynamic points in the center circle [11].

#### 2.2.2. Training Equipment

The VF training group wore the experimental tights and uniform training shoes with smart insoles and used the Podoon APP on the iPad Pro during training. The NVF training group wore the same experimental tights and training shoes without smart wearable devices.

### 2.3. Experimental Protocol

#### 2.3.1. Evaluation Protocol

Participants were recruited prior to the experiment, and their foot length was measured. Then, a smart foot pad matching their foot length was selected and cut. The participants had a five-minute warm-up run on a treadmill at 6 km/h to activate the lower extremity muscles for better balance control and one minute of rest. After the preparation, the thirty female college students were randomly divided into VFT, NVFT, or CG groups and completed tests before training as a pre-test. The pre-test consisted of 6 items, including one leg stance non-visual feedback (OLS-NF), one leg stance-visual feedback (OLS-VF), tandem stance (dominant leg in back)-non visual feedback (TSDL-NF), tandem stance (dominant leg in back)-visual feedback (TSDL-VF), tandem stance (non-dominant leg in back)-non visual feedback (TSNDL-NF), and tandem stance (non-dominant leg in back)-visual feedback (TSNDL-VF). Each item was measured three times, each time for 10 s. In order to ensure the consistency of the test, subjects in the study were all selected to have the right leg dominant. OLS is defined as the participant using the dominant leg to stand, while the non-supported leg was flexed at the knee with the plantar surface of the foot stabilized on the knee of the supporting leg [13]. TS is defined as the participant’s feet (on a line, heel-toe position) placed on the center of the force plate [14]. The iPad Pro with Podoon APP was located at an eye-level height, 1 m apart from the participants during the pre-test. At the end of the four weeks balance training, all participants performed the balance test again as a post-test to measure the effect of visual feedback on improving balance. The post-test was the same as the pre-test. 

#### 2.3.2. Training Protocol

After the pre-test, the participants underwent four weeks balance training, three times a week for 30 min each time, with 1-2 day intervals. Training included the VFT using Podoon APP to perform OLS/TS-VF balance training. The NVFT was given an open-eye balance training without Podoon APP participation on OLS/TS-NF. The control group did not do any training. The iPad Pro with Podoon APP was located at an eye-level height, 1 m apart from the participants. Participants in the VFT were asked to keep the dynamic point in the central circle as much as possible.

### 2.4. Sample Size Estimation

A priori power analysis (G*Power version 3.1.9.4; Heinrich Heine University Düsseldorf, Düsseldorf, Germany) showed that a minimum of 10 participants was required on the basis of conventional α (0.05) and β (0.80) values, with an effect size of 3.05. 

### 2.5. Statistical Analysis

In this study, the average values of three measurements for each item in each subject were calculated and used for statistical analysis. MATLAB (R2014a, The MathWorks Inc., Natick, MA, USA) was used for statistical analysis. The experiment used a mixed design two-way analysis of variance (ANOVA) (3 Group × 2 Times) to compare the differences between the pre-test and post-test of the three groups (VFT, NVFT, CG) in the six items: one leg stance non-visual feedback (OLS-NF), one leg stance-visual feedback (OLS-VF), tandem stance (dominant leg in back)-non visual feedback (TSDL-NF), tandem stance (dominant leg in back)-visual feedback (TSDL-VF), tandem stance (non-dominant leg in back)-non visual feedback (TSNDL-NF), and tandem stance (non-dominant leg in back)-visual feedback (TSNDL-VF). When the interaction was found to be significant, we used least significant difference comparisons between the three groups and *t*-test between the pre-test and post-test for post-hoc comparison. The level of significance was set at α < 0.05.

## 3. Results

After four weeks of balance training with different interventions, balance postures were assessed immediately after the training was completed. The position balance of VFT was significantly improved in OLS when compared with pre (*p* < 0.05). In addition, COP max displacement, COP velocity, COP radius, and COP area after intervention were significantly lower than that before intervention, except for the CG group. The effect from these interventions for the above COP parameter varied from weak to moderate across the balance conditions.

### 3.1. Analysis of COP_ML/AP_ Max Displacement 

**Figure 2:**
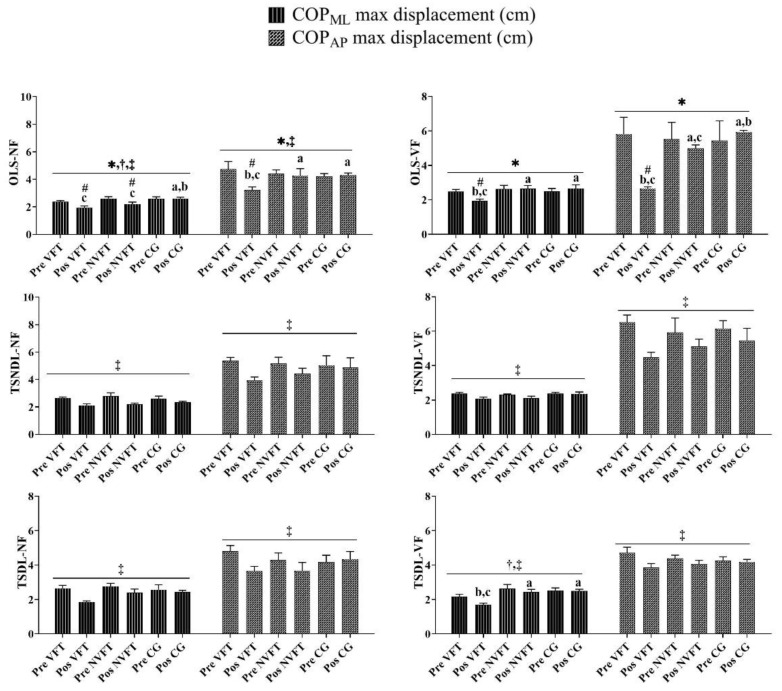
The training of group and time differences in the COP_ML_ and COP_AP_ maximum displacement parameters. Note: * Indicates a significant difference in interaction (Group*Times) (*p* < 0.05). ^†^ Indicates a significant difference in main effect (group) (*p* < 0.05). ^‡^ Indicates a significant difference in main effect (times) (*p* < 0.05). ^#^ Indicates a significant difference with pre-test. ^a^ Indicates a significant difference with VFT. ^b^ Indicates a significant difference with NVFT. ^c^ Indicates a significant difference with CG. VFT and NVFT before and after the test of various parameters of COP in OLS/TS. There was a significant difference in the interaction (Group*Times) of the COP_ML_ max displacement or COP_AP_ max displacement in OLS (*p* < 0.05). There was no significant difference in the interaction (Group*Times) of the COP_ML_ max displacement or COP_AP_ max displacement in TS (*p* > 0.05).

Figure 2 shows that significant interactions between Groups*Times were found in OLS-NF (*p* < 0.05). For the OLS-NF, post hoc analyses revealed that the COP_ML/AP_ max displacement of the visual feedback balance training group decreased after visual feedback balance training and the COP_ML_ max displacement of the non-visual feedback balance training group also decreased after traditional balance training. 

Figure 2 shows that significant interactions between Groups*Times were found in OLS-VF (*p* < 0.05). For the OLS-VF, post hoc analyses revealed that the COP_ML/AP_ max displacement of the visual feedback balance training group decreased after visual feedback balance training. 

Figure 2 shows that no significant interactions between Groups*Times were found for TSNDL-NF, TSNDL-VF, TSDL-NF, and TSDL-VF. There were time main effects for TSNDL-NF, TSNDL-VF, TSDL-NF, and TSDL-VF (*p* < 0.05). Post hoc analyses revealed that the COP_ML_/COP_AP_ max displacement between the pre-test and the post-test decreased in TSNDL-NF, TSNDL-VF, TSDL-NF, and TSDL-VF.

### 3.2. Analysis of COP_ML/AP_ Velocity 

**Figure 3:**
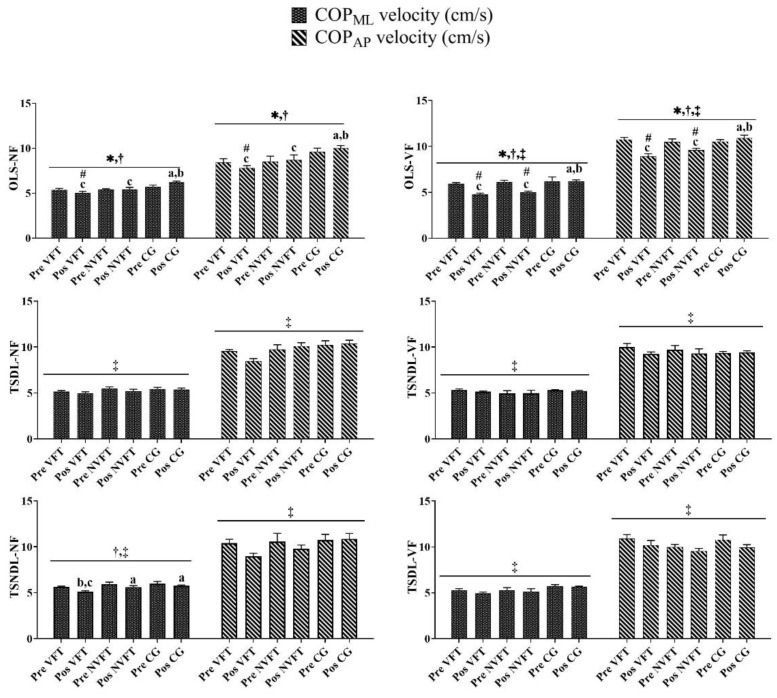
The training of group and time differences in the COP_ML_ and COP_AP_ velocity parameters. Note: * Indicates a significant difference in interaction (Group*Times) (*p* < 0.05). ^†^ Indicates a significant difference in main effect (group) (*p* < 0.05). ^‡^ Indicates a significant difference in main effect (times) (*p* < 0.05). ^#^ Indicates a significant difference with pre-test. ^a^ Indicates a significant difference with VFT. ^b^ Indicates a significant difference with NVFT. ^c^ Indicates a significant difference with CG. VFT and NVFT before and after the test of various parameters of COP in OLS/TS. There was a significant difference in the interaction (Group*Times) of the COP_ML_ velocity or COP_AP_ velocity in OLS (*p* < 0.05). There was no significant difference in the interaction (Group*Times) of the COP_ML_ velocity or COP_AP_ velocity in TS (*p* > 0.05).

Figure 3 shows that significant interactions between Groups*Times were found in OLS-NF (*p* < 0.05). For the OLS-NF, post hoc analyses revealed that the COP_ML/AP_ velocity of the visual feedback balance training group decreased after visual feedback balance training.

Figure 3 shows that significant interactions between Groups*Times were found in OLS-VF (*p* < 0.05). For the OLS-VF, post hoc analyses revealed that the COP_ML/AP_ velocity of the visual feedback balance training group decreased after visual feedback balance training; post hoc analyses revealed that the COP_ML/AP_ velocity of NVFT decreased after traditional balance training.

Figure 3 shows that no Significant interactions between Groups*Times were found in TSNDL-NF, TSNDL-VF, TSDL-NF, and TSDL-VF. There were time main effects in TSNDL-NF, TSNDL-VF, TSDL-NF, and TSDL-VF (*p* < 0.05). Post hoc analyses revealed that the COP_ML_/COP_AP_ velocity between the pre-test and the post-test decreased in TSNDL-NF, TSNDL-VF, TSDL-NF, and TSDL-VF.

### 3.3. Analysis of COP Radius and COP Area

**Figure 4:**
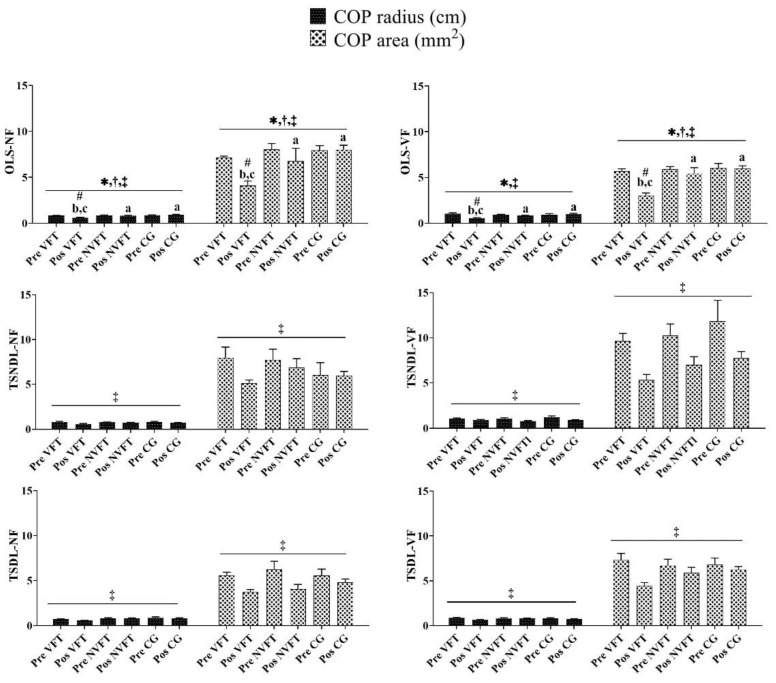
The training of group and times differences in the COP radius or COP area parameter. Note: * Indicates a significant difference in interaction (Group*Times) (*p* < 0.05). ^†^ Indicates a significant difference in main effect (group) (*p* < 0.05). ^‡^ Indicates a significant difference in main effect (times) (*p* < 0.05). ^#^ Indicates a significant difference with pre-test. ^a^ Indicates a significant difference with VFT. ^b^ Indicates a significant difference with NVFT. ^c^ Indicates a significant difference with CG. VFT and NVFT before and after the test of various parameters of COP in OLS/TS. There was a significant difference in the interaction (Group*Times) of the COP radius or COP area in OLS (*p* < 0.05). There was no significant difference in the interaction (Group*Times) of the COP radius or COP area in TS (*p* > 0.05).

Figure 4 shows that significant interactions between Groups*Times were found in OLS-NF (*p* < 0.05). For the OLS-NF, post hoc analyses revealed that the COP radius of the visual feedback balance training group and the COP area of the visual feedback balance training group decreased after visual feedback balance training. 

Figure 4 shows that significant interactions between Groups*Times were found in OLS-VF (*p* < 0.05). For the OLS-VF, post hoc analyses revealed that the COP radius of the visual feedback balance training group and the COP area of the visual feedback balance training group decreased after visual feedback balance training.

Figure 4 shows that no significant interactions between Groups*Times were found in TSNDL-NF, TSNDL-VF, TSDL-NF, and TSDL-VF. There were time main effects in TSNDL-NF, TSNDL-VF, TSDL-NF, and TSDL-VF (*p* < 0.05). Post hoc analyses revealed that the COP radius and COP area between the pre-test and the post-test decreased in TSNDL-NF, TSNDL-VF, TSDL-NF, and TSDL-VF.

## 4. Discussion

The purpose of this study was to explore balance training effects for healthy females, with a real-time VF technique of COP changes provided by smart wearable devices, on static balance postural control. After four weeks of balance training, the results showed that visual feedback training can improve healthy female stability of postural control by OLS and TS static balance training with a VFT intelligent app.

In this study, the decrease in COP_ML_ and COP_AP_ displacement with VFT demonstrated that the participants could control body sway in a considerably more stable manner with the help of real-time VF information. A past study had shown that real-time visual feedback can provide sensory information to the central nervous system (CNS), helping to reduce motor output variability [15]. Visual feedback balance training can enhance sensorimotor integration by a recalibration of the sensory systems [16]. In addition, the maintenance of body balance requires the joint action of CNS, vision, and somatosensory [17]. Therefore, after VF training in this study, the decrease in COP_ML_ and COP_AP_ max displacement may have been due to the increased proprioceptive integration ability and the stability of motor output with VF training. In addition, visual feedback balance training can increase muscle activity around the ankle and isokinetic muscle strength to improve balance ability [18]. Therefore, in this study, VF training may have increased ankle stability, thereby reducing COP displacement and improving balance. In summary, the decrease in AP and ML displacement of COP indicates that technical assistance training may increase balance control ability for reducing body swing and displacement changes after VF participation.

Previous studies have found that the smaller the displacement velocity, the better the balance control ability when using VF training [19]. In this study, VF training using smart auxiliary equipment may have helped subjects maintain better physical stability. When the human body is performing visual feedback training, the central nervous system controls the body’s goal-directed movements through relevant mechanisms [20]. The postural sway in the ML direction is controlled by adduction/abduction of the hip joint mechanism, while the posture sway in the AP direction is controlled by plantar flexion/dorsiflexion of the ankle joint mechanism [21]. Therefore, in this study, the decrease of the COP_ML_ velocity and the COP_AP_ velocity in the VFT may have been caused by the goal-directed movement of the ankle and hip joint mechanism, regulated by the CNS during VF training. In addition, past studies have found that balance training stimulates proprioception and increases sensory motor nerve signal transmission to improve balance control ability [18], while balance training also strengthens muscle activity and improves the stability of balance control [22]. Therefore, training without the assistance of smart devices will strengthen muscle activity. However, VFT gives visual feedback during the training process and the CNS controls the relevant muscle groups to perform goal-directed movements during training, so that the training effect of the visual feedback training group was higher than the general training group. In summary, the CNS mobilizes more motor neurons to increase the physical stability when performing VF training in OLS and TS.

Previous studies have shown that the COP radius and the COP area can reflect the static stability of the human body in the process of OLS; the larger the COP area and COP radius, the worse the stability [14]. Therefore, the results of this study show that balance training with visual feedback assisted by smart insoles can help subjects maintain better physical stability. The decrease in COP radius and COP area is mainly due to the conscious control by the human body, based on the visual information obtained from VF [23]. During the training process, the subjects could integrate VF information and motor sensory information to maintain physical stability under the control of the CNS [24]. Therefore, the decrease of COP area and COP radius after training with VF provided by smart insoles may have been due to the increase of visual information. In addition, past studies have pointed out that smart wearable devices give VF to the body’s COM, and the COM VF will strengthen autonomous control and reduce postural sway, thereby achieving more efficient posture control or improving balance [9]. The training without smart auxiliary equipment only adjusts using the original sense organ system, and cannot judge the position effectively through VF [25]. Therefore, the balance ability of the NVFT cannot be significantly improved, and the use of VF assisted by smart insoles for training provides more VF information to strengthen the physical autonomous control ability and improve the physical balance ability.

A limitation of this study is that the experiment was conducted in a small sample of participants, and the small amount of training the participants received may not have been sufficient to measure the adaptation effect of the training. Therefore, although the value of COP of VFT after training was higher than CG/NVFT, the training effect needs more samples for further verification. In addition, the study only assessed the static standing of female subjects in stable postures, and no further discussion was made of postural balance with an unstable plane/external disturbance or the involvement of male subjects; our future research will address these issues.

## 5. Conclusions

Visual feedback training may increase the participants’ visual information and promote the integration of the central nervous system, thereby assisting proprioception to reduce the center of pressure movement and enhance balance ability. In this study, the healthy females increased their balance ability after visual feedback training and found that the need for visual feedback in the balanced posture on one foot was significantly higher than that with two feet. It is particularly important to use smart wearable devices to improve the body’s ability to maintain balance.

## Data Availability

The datasets used and analyzed in the current study are included in this article.

## Abbreviations

COP: center of pressure; VFT: visual feedback balance training group; NVFT: non-visual feedback balance training group; CG: control group; COPAP: COP anteroposterior; COPML: COP mediolateral; COM: center of mass; OLS: one leg stance; TS: tandem stance; OLS-NF: one leg stance non-visual feedback; OLS-VF: one leg stance-visual feedback; TSDL-NF: tandem stance (dominant leg in back)-non visual feedback; TSDL-VF: tandem stance (dominant leg in back)-visual feedback; TSNDL-NF: tandem stance (non-dominant leg in back)-non visual feedback; TSNDL-VF: tandem stance (non-dominant leg in back)-visual feedback; CNS: central nervous system.
